# Core Needle Biopsy (CNB) and Immunohistochemical Markers in Indeterminate Thyroid Nodules: Evidence and Perspectives

**DOI:** 10.2174/0118715303433603251129105835

**Published:** 2026-04-13

**Authors:** Salvatore Monti, Valerio Renzelli, Beatrice Fazzalari, Claudia Bongermino, Maria Grazia Deiana, Giuseppe Pugliese

**Affiliations:** 1 Endocrinology and Diabetology Unit, Azienda Ospedaliera Sant’Andrea, Via di Grottarossa, Rome 1035 - 00189 , Italy;; 2 Freelance Doctor, Diabetologist and Endocrinologist, Rome 1035 - 00189, Italy

**Keywords:** Core needle biopsy, thyroid, CNB, HBME1, Galectin-3, CK19, CD56

## Abstract

Thyroid nodules are a prevalent endocrine condition that requires accurate diagnostic tools to guide appropriate clinical management. Despite advances in imaging and cytology, evaluating thyroid nodules remains challenging, particularly in cases with indeterminate cytology. This narrative review examines the effectiveness of core needle biopsy (CNB) as a diagnostic approach, with a specific focus on its integration with immunohistochemical markers. CNB offers high diagnostic accuracy that improves with operator experience. The inclusion of immunohistochemical markers (CK19, CD56, Galectin-3, and HBME1) further enhances diagnostic precision, particularly in stratifying indeterminate nodules. However, CNB cannot reliably distinguish follicular adenoma from follicular carcinoma preoperatively because diagnosis requires evaluation of complete capsular and/or vascular invasion on the surgical specimen, which remains a fundamental limitation for follicular-patterned nodules. Despite its accuracy, broader adoption remains limited by non-standardized CNB protocols, inter-observer variability for IHC interpretation, and heterogeneous reimbursement policies across health systems, especially outside Western contexts. Comparative data on patient-reported outcomes and local cost-effectiveness in Asian and Indian settings are still variable and require multicenter validation before routine implementation can be generalized. The findings of this review reinforce the growing evidence that supports CNB as an effective diagnostic tool for thyroid nodules, especially when combined with immunohistochemical markers. This approach has the potential to improve diagnostic accuracy, reduce unnecessary surgical interventions, and enhance patient outcomes. Future research should focus on standardizing procedures and conducting large-scale validation studies.

## 1 INTRODUCTION

Fine-needle aspiration (FNA) plays a crucial role in the diagnostic evaluation of patients with thyroid nodules. It is safe, effective, and cost-efficient. However, clinical experience has revealed several limitations that require improvement. Thyroid cancer diagnosis relies on different classification systems for reporting cytologic results, which are central to interpreting specimens. Many classification systems have been proposed by various societies. The Bethesda System is one of the most widely used globally, while in Italy, the Italian Consensus for the Classification and Reporting of Thyroid Cytology (ICCRTC) is preferred [1].

In the Bethesda system [2], FNA results showing atypia of undetermined significance/follicular lesion of undetermined significance (AUS/FLUS, category III) or follicular neoplasm/suspicious for a follicular neoplasm (FN/SFN, category IV) are classified as indeterminate. In the Italian Consensus, indeterminate nodules are included in the TIR3A and TIR3B categories, while in the UK Royal College of Pathologists guidelines, they are classified as Thy 3a (‘a’ for atypia) and Thy 3f (‘f’ for follicular) [3]. The risk of malignancy for these categories, based on surgical resection data, ranges from 13 to 35% [2].

Additionally, FNA samples may be insufficient for in- depth tests that could improve diagnostic accuracy, especially for indeterminate nodules [4]. Consequently, these indeterminate cytological categories often fail to provide a definitive diagnosis, necessitating further testing or surgery. Thyroid surgery carries a relatively high complication rate, even though most indeterminate nodules and follicular neoplasms are benign. Further diagnostic methods are therefore required to prevent unnecessary surgery and associated morbidity [5].

Given these limitations, core needle biopsy (CNB) has become an important tool for evaluating thyroid nodules. CNB is considered highly reliable for assessing nodules and identifying patients who may require surgery. The histological specimens obtained *via* CNB are generally superior to cytological material. Therefore, CNB can complement FNA in difficult or unclear cases of thyroid neoplasia with uncertain malignant potential [6].

CNB is rarely used as the initial procedure due to its slightly higher risk of bleeding and increased patient discomfort. It is typically recommended after one or more nondiagnostic FNA [7]. Unlike FNA, which samples multiple areas of a nodule, CNB provides a single core sample. However, a repeat CNB may yield more detailed information about thyroid architecture in lesions categorized as indeterminate [8]. CNB may also be more useful than FNA for suspected lymphoma. Hence, CNB is generally considered complementary to FNA rather than a first-line diagnostic approach.

Because CNB provides larger tissue samples, immunohistochemistry (IHC) or molecular testing can often be performed on additional paraffin-embedded sections. A combination of IHC markers is commonly used to diagnose thyroid carcinoma [6]. Markers such as Galectin-3, HBME-1, Cytokeratin 19 (CK19), and CD56 are widely applied because they improve diagnostic accuracy [9]. Although these markers have been studied since the mid-20th century, their use is not yet fully standardized.

Individually, these markers have limited accuracy, as they may also be expressed in benign lesions [9, 10]. Galectin-3 is involved in cell migration, adhesion, and apoptosis, and is expressed primarily in papillary thyroid carcinoma (PTC) but is usually absent in benign lesions [9, 11-13]. Among these markers, Galectin-3 demonstrates the highest diagnostic accuracy [14]. CK19 is widely expressed in PTC but shows heterogeneous expression in follicular carcinomas [13, 15]. Retrospective analyses have shown a positive correlation between CK19 and Galectin-3 expression in PTC [16]. HBME-1 is strongly expressed in differentiated thyroid carcinomas, both follicular and non-follicular [15]. CD56, a cell adhesion molecule, is normally expressed by thyroid cells, and its loss indicates malignant potential. Its expression is reduced or lost in papillary, follicular, and anaplastic carcinoma [17].

Molecular diagnostics can provide an additional option for cytologically indeterminate nodules. However, molecular tests currently marketed in the USA are not reimbursed outside the country due to differences in healthcare systems, lack of independent validation, absence of long-term outcome data, and high costs. Asian consensus statements [18] have positioned CNB as a complementary method to reduce nondiagnostic rates and clarify indeterminate categories, with detailed recommendations on indications, technique, reporting, and safety validated in large Korean cohorts. In Chinese comparative studies [19], CNB demonstrated a higher diagnostic yield than repeat FNA, with mostly minor, compressible bleeding and no increase in major complications, supporting its clinical acceptability beyond Western settings. Low-cost, locally developed NGS workflows for indeterminate thyroid nodules are also emerging in India [20], suggesting growing integration of histology, IHC, and genomics in resource-constrained settings.

## 2 METHODOLOGY

This narrative review provides a comprehensive overview of the role of core needle biopsy (CNB) and the biomarkers CK19, CD56, Galectin-3, and HBME1 in diagnosing indeterminate thyroid nodules. A literature search was conducted across multiple databases, including PubMed, Scopus, and Web of Science, focusing on articles published between January 1981 and July 2025. Keywords such as “core needle biopsy”, “thyroid nodules ”, “CK19” , “CD56”, “Galectin-3”, and “HBME1” were used to identify relevant studies.

This review is narrative in nature and was not preregistered, nor did it follow a PRISMA-driven protocol. Studies were included based on clinical relevance, methodological clarity, and representativeness of current practice. Selection and synthesis were performed narratively to integrate heterogeneous study designs and endpoints that preclude formal meta-analysis, with the explicit aim of generating clinically actionable insights and highlighting areas for future standardized research. The decision to adopt a narrative approach was driven by the substantial heterogeneity of available studies and the lack of randomized controlled trials on this topic. Unlike a systematic review, this approach relies on targeted searches, pragmatic inclusion, qualitative synthesis, and no preregistered protocol or quantitative pooling, prioritizing clinical interpretability over exhaustive enumeration.

Studies were selected based on their relevance to the diagnostic accuracy and clinical utility of CNB and the specified biomarkers. Both original research articles and review papers were considered. The literature was analyzed and synthesized descriptively, emphasizing interpretation of findings and identification of emerging trends and gaps in current knowledge.

Through this narrative approach, we aim to integrate diverse perspectives and provide a holistic understanding of the current state of research on CNB and biomarkers in indeterminate thyroid nodules.

## 3 DISCUSSION

### 3.1 Core Needle Biopsy (CNB)

To overcome indeterminate or inconclusive results from FNA, core needle biopsy (CNB) has been proposed as a complementary or alternative procedure for further evaluation of thyroid nodules. Since its introduction in the 1990s [21], CNB has demonstrated reliability and safety while improving the diagnosis of thyroid carcinomas [22]. The CNB procedure typically uses a large-bore, spring-activated needle (usually 16–18G) to obtain a histologic sample of the nodule. This approach provides the pathologist with tissue architecture and enables immunohistochemical staining [23], further increasing diagnostic yield.

Despite the use of larger-bore needles, complication rates are low, especially when performed by experienced operators. A meta-analysis of 6,687 nodules reported major complication rates of 0.06% and minor complication rates of 0.76% [24]. Major complications included massive hematoma, pseudoaneurysm, and voice changes lasting more than 30 days, whereas minor complications included small-to- moderate hematoma (most frequent), carotid injury, transient voice changes, tracheal puncture, and dysphagia. Rare adverse events such as edema, vertebral puncture, and vasovagal reactions were also recorded. CNB using thinner gauges (21G) has been proposed and is currently used, yielding similar diagnostic results to larger needles without increasing adverse effects compared to FNA [25]. Prospective patient-reported outcomes indicate no significant differences between CNB and FNA in intraprocedural or early postprocedural pain scores or overall satisfaction at two-week follow-up, with no major complications in either group [26]. Contemporary guidelines similarly report comparable pain and tolerability to FNA when 18–21G devices are used, with hematoma being the most common event, usually managed by compression [5, 27].

Multiple meta-analyses and retrospective studies have demonstrated the diagnostic usefulness of CNB for indeterminate thyroid nodules. For example, Su *et al.* [19] reported higher diagnostic accuracy for CNB compared to FNA (70.7% *vs*. 35.8%) and greater patient satisfaction (83.9% *vs*. 66.8%). These findings are consistent with previous reports by Joo *et al.* [28], Hong *et al.* [29], Trimboli *et al.* [30], Suh *et al.* [8, 31], and Hahn *et al.* [32].

Repeated CNB of the same nodule can further improve diagnostic accuracy and reduce unnecessary thyroidectomies. A retrospective study in Korea [33] on nodules previously classified as atypia of undetermined significance with architectural atypia showed that repeated CNB did not increase malignancy detection but reclassified 30.2% of nodules as benign, reducing unnecessary surgery.

CNB is also valuable in diagnosing thyroid lymphoma. While FNA may not clearly differentiate lymphoma from thyroiditis—a common cytological background—CNB can overcome this limitation and facilitate accurate diagnosis [34].

From a cost perspective, CNB represents a valid alternative to FNA. Trimboli *et al.* [35] demonstrated that CNB reliably detects benign nodules previously classified as indeterminate by FNA while reducing costs by approximately 33.8% compared to diagnostic thyroidectomy. Western studies report similar savings (~30–35%), whereas Asian analyses highlight that device selection and market pricing affect cost-benefit, emphasizing the need for local economic evaluations [36]. In India, the development of low-cost NGS workflows [20] suggests a complementary strategy to IHC-heavy algorithms, with method selection guided by laboratory capacity and per-test costs.

Overall, CNB is a reliable complementary and alternative technique for thyroid nodule evaluation, minimizing unnecessary surgery with a low risk of complications. However, a fundamental limitation remains: CNB cannot preoperatively distinguish follicular adenoma from follicular carcinoma, as definitive diagnosis requires histologic demonstration of capsular and/or vascular invasion across the entire tumor- capsule interface, which cannot be reliably assessed in a core sample [37].

### 3.2 Immunohistochemical Markers

The combined expression patterns of immunohistochemical (IHC) markers can further assist in the diagnosis of thyroid cancer. IHC is widely employed in routine thyroid pathology. An integrated assessment of CK19, CD56, Galectin-3, and HBME-1 serves as an indispensable adjunct to conventional histology, helping to clarify differential diagnoses in histological specimens obtained from thyroidectomy (Table **1**) [10].

### 3.3 CK19

Cytokeratin-19 (CK19) is an intermediate filament protein (~40 kDa) that contributes to the structural integrity of epithelial cells. CK19 is strongly and diffusely expressed in papillary thyroid carcinoma (PTC), whereas it is weakly expressed or absent in benign thyroid lesions, making it a valuable marker for distinguishing between malignant and benign lesions [13].

In PTC, CK19 exhibits intense and diffuse expression in both cytological and surgical specimens. In contrast, follicular thyroid carcinomas (FTC) show variable expression, while follicular adenomas (FA) display localized or absent CK19 staining [14]. Mukherjee *et al.* [38] reported that CK19 expression in papillary carcinoma has a specificity ranging from 75% to 97%. Other studies have shown similar findings: Liberman *et al.* [39] observed uniform CK19 reactivity in 100% of PTC cases, Cheung *et al.* [40] reported diffuse CK19 staining in 80% of typical PTC variants, and Zhu *et al.* [41] concluded that CK19 is useful not only for differentiating benign from malignant papillary structures but also for distinguishing PTC from FTC. They noted that CK19 staining in PTC papillae is strong and diffuse, while in benign tissues it is weak and focal. CK19 expression is significantly higher in follicular PTC than in FTC. Medullary and undifferentiated thyroid carcinomas were negative for CK19 [38].

Elsers *et al.* [42] demonstrated that CK19 is expressed in malignant follicular-patterned thyroid lesions with high sensitivity, specificity, and negative predictive value. These findings are consistent with previous reports documenting strong and diffuse CK19 staining in the follicular variant of PTC (FVPTC) and FTC [14, 43-45]. Although CK19 may also be detected in some hyperplastic nodules, FA, and non-invasive follicular thyroid neoplasms with papillary-like nuclear features (NIFTP), staining in these cases is typically weak, focal, and patchy [41, 46].

### 3.4 CD56

CD56 is a neural cell adhesion molecule involved in regulating cell motility and limiting tumor invasion. It is normally expressed in thyroid follicular cells, where it is associated with cellular differentiation, but its expression is decreased or absent in malignant thyroid lesions [14, 43]. Loss of CD56 expression has been reported in papillary [47], follicular, and anaplastic thyroid carcinomas [14, 48]. Several studies indicate that CD56 loss correlates with higher metastatic potential and poorer prognosis in various malignancies, including thyroid cancer [17].

Unlike other immunohistochemical markers, CD56 positivity generally indicates benign thyroid tissue. A meta-analysis by Pyo *et al.* demonstrated that PTC and FTC exhibit a higher rate of CD56 loss compared to benign lesions, such as follicular adenomas (FA), benign follicular nodules, Hashimoto’s thyroiditis, and normal thyroid tissue. Consequently, CD56 immunohistochemistry can be a useful tool for differentiating follicular variant PTC (FVPTC) and FTC from FA [49].

Among the commonly used thyroid markers, CD56 loss may have greater specificity in identifying malignancy [14, 17, 48]. However, in a case series of 18 follicular tumors with uncertain malignant potential, some lesions co-expressed both CD56 and Galectin-3, indicating that no single marker is fully definitive [50].

### 3.5 Galecin-3

Galectin-3 is a 31-kDa protein belonging to the galectin family, capable of interacting with both intracellular glycoproteins and extracellular matrix proteins. It is the only galectin with a pentameric structure, which enables the formation of cross-linked glycoprotein networks at the cell surface, influencing cellular signaling and receptor endocytosis [51]. Gal-3 is expressed in the nucleus, cytoplasm, and extracellular space, and plays roles in regulating apoptosis, cell growth, and tumor progression [52].

Gal-3 evaluation is frequently used to aid in the assessment of cytologically indeterminate thyroid nodules, as several studies have demonstrated its high accuracy in predicting malignancy, particularly in classical PTC [11]. Bartolazzi *et al.*, in one of the earliest studies involving around 1,000 thyroid nodules, reported that Gal-3 is a reliable marker for distinguishing thyroid carcinoma from benign lesions, with good sensitivity and specificity [12].

However, Gal-3 expression is not entirely specific to malignancy, as it may also be detected in thyroiditis, goiters, and follicular adenomas [53]. A meta-analysis including 8,172 thyroid nodules reported that Gal-3 was positive in 87% of histologically confirmed thyroid cancers, while 87% of benign lesions were negative, confirming its diagnostic utility [54].

In a cohort of 432 thyroid follicular proliferations with indeterminate cytology, preoperative immunohistochemical evaluation of Gal-3 correctly classified 88% of the nodules, highlighting its potential to reduce unnecessary thyroid surgeries. Nevertheless, approximately 22% of Gal-3-negative cancers could be missed [55].

Among the commonly used markers, Gal-3 appears to have the highest diagnostic accuracy for thyroid malignancy [14].

### 3.6 HBME1

Hector Battifora Mesothelial-1 (HBME-1) is a monoclonal antibody directed against an unidentified antigen present on the microvilli of mesothelial cells, and is also expressed in adenocarcinomas of the lung, breast, pancreas, and thyroid [56]. Currently, its primary application is in the diagnosis of thyroid tumors.

HBME-1 expression varies depending on the tumor type. While it may be negative in undifferentiated thyroid carcinomas, it is widely expressed in differentiated thyroid carcinomas [15]. Nonetheless, HBME-1 positivity can also be observed in certain benign conditions, such as adenomatous goiter and follicular adenomas [57].

For malignant thyroid nodules, HBME-1 demonstrates a sensitivity of 77% and a specificity of 83%, with a higher prevalence in tumors of follicular origin [58]. The marker has gained additional significance with the recognition of non-invasive follicular neoplasms, which show HBME-1 positivity in approximately 78% of cases, particularly in follicular nodules with indeterminate cytology [59].

Although HBME-1 alone has relatively low sensitivity, combining it with other markers substantially improves diagnostic performance. For example, its association with Galectin-3 increases sensitivity to 94% and achieves a negative predictive value of 92% [59]. A recent systematic review of pre-surgical markers confirmed that both Gal-3 and HBME-1 have high specificity (86% and 82%, respectively) but moderate sensitivity (76% and 75%) for distinguishing malignant from benign thyroid nodules [13].

### 3.7 Emerging IHC Markers

TROP-2 overexpression is significantly associated with BRAF V600E in papillary thyroid carcinoma, and TROP-2 immunohistochemistry may aid in predicting BRAF status and refining differential diagnosis alongside CK19, HBME-1, Gal-3, and CD56 in CNB specimens [60, 61]. Selected series and pathology-oriented reviews support the adjunctive value of Ki-67 [18] quantification and p63 in specific diagnostic panels, particularly for challenging papillary-like and follicular-patterned lesions, complementing classic markers and targeted BRAF testing in discordant profiles [17, 43, 62-64].

### 3.8 CNB and Immunohistochemical Markers: Increasing the Diagnostic Threshold

In cases where initial FNA results are nondiagnostic or indeterminate, CNB offers several advantages over repeat FNA. Importantly, CNB does not preclude the use of ancillary techniques, such as immunohistochemistry (IHC) and molecular testing, which provide clinicians with more comprehensive diagnostic information on the sampled nodule [65]. Numerous studies have investigated the utility of IHC markers in CNB specimens classified as indeterminate, with the aim of improving diagnostic accuracy, guiding appropriate management, reducing unnecessary surgeries, and optimizing patient outcomes [66].

The rate of unsatisfactory results after applying IHC markers is markedly lower than with CNB alone [66]. For example, Xiong *et al.* demonstrated that in approximately 13% of CNB samples, malignancy could not be determined based solely on morphology and required IHC evaluation [67]. Single biomarkers generally lack sufficient sensitivity or specificity to reliably distinguish benign from malignant thyroid lesions. Consequently, combining multiple markers is a more effective strategy. Combined immunopositivity for Galectin-3, CK19, and HBME-1 achieves high sensitivity (95%) and specificity (97%) for the diagnosis of papillary thyroid carcinoma (PTC) [44, 53, 68].

Arcolia *et al.* reported that co-expression of Gal-3 and CK19 yielded 92% sensitivity and 99% specificity, while Gal-3 combined with HBME-1 provided 95% sensitivity and 95% specificity for PTC [44]. It should be emphasized, however, that the expression of HBME-1, Gal-3, and CK19 is not predictive of tumor aggressiveness and should not be used to guide the extent of surgical excision [10].

Certain variants of PTC, such as the follicular variant, may present diagnostic challenges due to subtle nuclear features or a predominant follicular architecture. Moreover, some benign lesions—including Hashimoto’s thyroiditis, multinodular goiter, follicular or oncocytic adenomas, and lesions with degenerative changes—may mimic PTC cytologically, with features such as nuclear grooves and chromatin clearing [69, 70].

Among individual markers, CD56 has emerged as a particularly useful tool in immunohistochemistry panels for thyroid epithelial neoplasms. Normally expressed in thyroid follicular cells, including benign conditions such as goiter, Graves’ disease, and Hashimoto’s thyroiditis, CD56 is consistently absent in PTC, including its follicular variants [17, 71]. Park *et al.* reported CD56 positivity in 93% of benign lesions but only 5% of malignant lesions, highlighting its role as a negative diagnostic marker for PTC [71]. Similarly, EI Demellawy *et al.* observed diffuse CD56 expression in normal follicular epithelium but not in PTC, concluding that CD56 is a reliable discriminator of PTC from other neoplasms of follicular origin, with sensitivity and specificity reaching 100% [17]. Shahebrahimi *et al.* and Shin *et al.* confirmed these findings, demonstrating CD56 expression in 93% of benign lesions and only 5–7% of malignant lesions, with sensitivity and specificity up to 94.8% and 92.3%, respectively [72, 73].

The combined application of multiple markers further improves diagnostic accuracy. When Gal-3, CK19, HBME-1, and CD56 are evaluated together, studies report 87.5% sensitivity, 100% specificity, and 100% positive predictive value for distinguishing PTC from benign thyroid lesions [73]. Song *et al.* similarly demonstrated that Gal-3 and CK19 provide high specificity (96.4% and 100%, respectively), HBME-1 offers high sensitivity (87.5%), and CD56 contributes moderate sensitivity and specificity (60% and 72%) for identifying benign nodules, reinforcing the need for a panel approach [66].

Notably, CNB specimens provide superior material for IHC compared with FNA. In a direct comparison, the rate of continued nondiagnosis was significantly lower in CNB samples than FNA (11.3% *vs*. 42.9%), resulting in a substantially higher probability of lesion reclassification and more accurate preoperative diagnosis [66].

### 3.9 Clinical Implications and Areas of Research

The management of indeterminate thyroid nodules remains a significant clinical challenge. A substantial proportion of these nodules ultimately lead to unnecessary surgeries, exposing patients to procedural risks and generating considerable economic burden for healthcare systems. Therefore, accurate and minimally invasive diagnostic tools are essential to reduce unnecessary interventions, optimize patient outcomes, and improve overall satisfaction.

Core needle biopsy (CNB), particularly when combined with immunohistochemical (IHC) markers, has emerged as a promising strategy in this context. Although CNB is still relatively novel in thyroid nodule evaluation, accumulating evidence demonstrates that it enhances diagnostic accuracy compared to FNA, particularly in nodules initially classified as indeterminate. Furthermore, the integration of IHC markers—such as CD56, Galectin-3, HBME1, and CK19—can further improve both the sensitivity and specificity of CNB. This combined approach aids in distinguishing malignant from benign nodules and reduces the number of inconclusive results, allowing clinicians to stratify malignancy risk more effectively. Consequently, high-risk patients can be prioritized for surgical intervention, while low-risk patients may be managed conservatively.

However, several challenges hinder the widespread clinical implementation of CNB and IHC. First, CNB requires operators with specific training and experience, which may be limited in smaller or resource-constrained healthcare settings. Establishing training programs demands both time and financial investment. Second, IHC testing remains costly and is often not reimbursed by insurance or publicly funded healthcare systems. As a result, some patients may forego these diagnostic procedures or opt directly for surgery, which is often fully covered, despite its higher invasiveness and risk. When evaluating costs, CNB with IHC should not be compared with FNA alone, but rather with the alternative therapeutic options—diagnostic surgery and molecular testing. Considering both the economic and health-related costs of surgery, CNB with IHC offers a favorable cost-benefit profile.

Another major limitation is the lack of standardization in IHC use. No universally accepted diagnostic panel or threshold for marker positivity currently exists, introducing variability in interpretation and limiting adoption in routine practice. Additionally, CNB may be technically challenging for nodules that are small, deep, or located near critical structures such as the thyroid capsule, carotid artery, or trachea.

A fundamental limitation of CNB remains the evaluation of follicular thyroid nodules. Indeterminate cytology often includes follicular lesions, but distinguishing follicular adenoma from follicular carcinoma requires histological assessment of capsular and vascular invasion—features that cannot be reliably evaluated preoperatively using CNB. Nevertheless, follicular nodules account for approximately 20–40% of indeterminate nodules, while follicular carcinoma represents only 12–15% of these lesions [74, 75]. Moreover, among malignant nodules within the indeterminate category, only 10–20% are follicular carcinomas, with the majority being PTCs, particularly classical and follicular variants [74-77]. Therefore, CNB, particularly when combined with IHC markers, offers high diagnostic accuracy for the majority of indeterminate non-follicular nodules and may also help identify malignant forms within follicular lesions.

Despite these limitations, CNB combined with IHC holds significant potential for refining the diagnostic evaluation of indeterminate thyroid nodules. Large-scale prospective studies are needed to validate these findings, establish standardized protocols, and assess cost-effectiveness across diverse healthcare settings.

Future research should also explore the integration of advanced technologies, including omics (genomics, proteomics, metabolomics) and artificial intelligence, to further enhance diagnostic precision. Digital pathology and AI-based image analysis may reduce inter-observer variability by standardizing the interpretation of IHC patterns (*e.g.*, CK19, HBME1, Galectin-3, CD56, TROP2) and Ki-67 quantification, allowing for more reproducible reporting across centers. When combined with targeted genomic analyses (*e.g.*, BRAF, RET, RAS) and potentially transcriptomic or proteomic profiling, such approaches could provide decision support for triaging Thy3/TIR3 nodules and identifying patients who truly require surgery [78]. Multicenter datasets from both Asian and European institutions will be essential to train and externally validate these models before routine clinical implementation. Ultimately, these integrated diagnostic strategies have the potential to deliver a more personalized and precise approach to thyroid nodule management (Fig. **1**).

## CONCLUSION

The evidence reviewed highlights the growing recognition of core needle biopsy (CNB) as a pivotal diagnostic tool for managing thyroid nodules with indeterminate cytology. As a minimally invasive procedure, CNB addresses several limitations of fine-needle aspiration (FNA), particularly in cases where cytological results are inconclusive. Following indeterminate cytology, CNB demonstrates a higher diagnostic yield than repeat FNA, while maintaining comparable patient-reported pain and satisfaction. Major complications are rare when procedures are performed by experienced operators.

The diagnostic performance of CNB can be further enhanced through the use of combined immunohistochemical panels, including HBME-1, Galectin-3, CK19, and CD56, with the addition of TROP-2 and reflex BRAF testing in discordant cases. This integrated approach improves nodule reclassification and reduces residual preoperative uncertainty. Successful implementation of CNB should be supported by operator training, locally adapted cost-sensitive workflows, and, where feasible, digital pathology and artificial intelligence to standardize interpretation and improve reproducibility.

Despite these advantages, certain challenges limit broader adoption. The absence of universally accepted cut-off values for immunohistochemical markers introduces variability in interpretation, and inter-observer differences in assessing marker expression further complicate consistency across centers. Standardized protocols, rigorous training, and consensus on marker thresholds are therefore essential to maximize diagnostic accuracy and clinical utility.

Future research should focus on addressing these limitations. Large-scale, multicenter validation studies are critical to confirm the reproducibility and clinical applicability of CNB combined with immunohistochemistry across diverse populations. Furthermore, the development of integrated diagnostic algorithms that incorporate CNB, immunohistochemical, and molecular data could provide a systematic and evidence-based framework for managing indeterminate thyroid nodules, minimizing unnecessary surgery, and improving patient outcomes.

## Figures and Tables

**Fig. (1):**
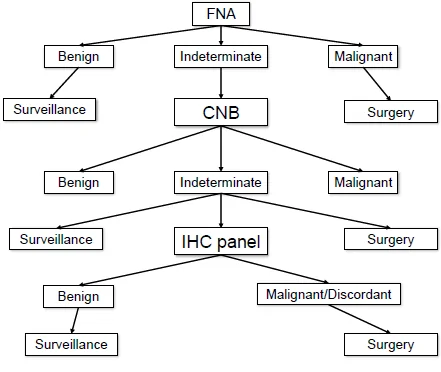
Diagnostic workflow.

**Table 1:** Immunohistochemical markers.

Marker	-
CK19	Intense and diffuse expression in PTC and absent or faintly expressed in benign thyroid lesions.
CD56	Expression in normal thyroid follicular cells and relates to their differentiation, with reduced/absent expression in malignant thyroid lesions; loss of expression is associated with metastatic potential and poor prognosis.
Galectin-3	Expressed in carcinomas, especially classic PTC, but also in thyroiditis, goiters and follicular adenomas.
HBME1	Highly expressed in differentiated thyroid carcinomas but may be partially or negatively expressed in undifferentiated thyroid carcinoma; may be positive in benign nodules.

**Abbreviations:** PTC: papillary thyroid carcinoma.

## LIST OF ABBREVIATIONS

FNA: Fine-Needle Aspiration
ICCRTC: Italian Consensus for the Classification and Reporting of Thyroid Cytology
CK19: Cytokeratin-19
PTC: Papillary Thyroid Carcinoma
FTC: Follicular Thyroid Carcinoma
FA: Follicular Adenoma
IHC: Immunohistochemistry

## References

[1] Nardi F., Basolo F., Crescenzi A., Fadda G., Frasoldati A., Orlandi F., Palombini L., Papini E., Zini M., Pontecorvi A., Vitti P. (2014). Italian consensus for the classification and reporting of thyroid cytology.. J. Endocrinol. Invest..

[2] Ali S.Z., Baloch Z.W., Cochand-Priollet B., Schmitt F.C., Vielh P., VanderLaan P.A. (2023). The 2023 bethesda system for reporting thyroid cytopathology.. Thyroid.

[3] (2024). Guidance on the reporting of thyroid cytology specimens.. https://www.rcpath.org/static/d40af26c-0e49-4d40-8fce4a38f2a5c44b/G089-Guidance-on-the-reporting-of-thyroid-cytology-specimensFor-Publication.pdf.

[4] Sakorafas G.H. (2010). Thyroid nodules; Interpretation and importance of fine-needle aspiration (FNA) for the clinician – Practical considerations.. Surg. Oncol..

[5] Karatay E., Javadov M., Kaya H. (2023). Thyroid nodule core needle biopsy — current approach.. Endokrynol. Pol..

[6] Dolidze D.D., Covantsev S., Chechenin G.M., Pichugina N.V., Bedina A.V., Bumbu A. (2024). Core needle biopsy for thyroid nodules assessment-a new horizon?. World J. Clin. Oncol..

[7] Wang C., Vickery A.L., Maloof F. (1976). Needle biopsy of the thyroid.. Surg. Gynecol. Obstet..

[8] Suh C.H., Baek J.H., Park C., Choi Y.J., Lee J.H. (2017). The role of core needle biopsy for thyroid nodules with initially indeterminate results on previous fine-needle aspiration: A systematic review and meta-analysis.. AJNR Am. J. Neuroradiol..

[9] Saggiorato E., De Pompa R., Volante M., Cappia S., Arecco F., Dei Tos A.P., Orlandi F., Papotti M. (2005). Characterization of thyroid ‘follicular neoplasms’ in fine-needle aspiration cytological specimens using a panel of immunohistochemical markers: A proposal for clinical application.. Endocr. Relat. Cancer.

[10] Crescenzi A., Baloch Z. (2023). Immunohistochemistry in the pathologic diagnosis and management of thyroid neoplasms.. Front. Endocrinol..

[11] Sciacchitano S., Lavra L., Ulivieri A., Magi F., Porcelli T., Amendola S., De Francesco G.P., Bellotti C., Trovato M.C., Salehi L.B., Bartolazzi A. (2016). Combined clinical and ultrasound follow-up assists in malignancy detection in Galectin-3 negative Thy-3 thyroid nodules.. Endocrine.

[12] Bartolazzi A., Gasbarri A., Papotti M., Bussolati G., Lucante T., Khan A., Inohara H., Marandino F., Orlandi F., Nardi F., Vecchione A., Tecce R., Larsson O. (2001). Application of an immunodiagnostic method for improving preoperative diagnosis of nodular thyroid lesions.. Lancet.

[13] Mohan U., Sunny S.P., Mendonca P., Kuriakose M.A., Kannan S., Suresh A. (2022). Systematic review and meta-analysis to identify the immunocytochemical markers effective in delineating benign from malignant thyroid lesions in fnac samples.. Endocr. Pathol..

[14] Dunđerović D., Lipkovski J.M., Boričic I., Soldatović I., Božic V., Cvejić D., Tatić S. (2015). Defining the value of CD56, CK19, Galectin 3 and HBME-1 in diagnosis of follicular cell derived lesions of thyroid with systematic review of literature.. Diagn. Pathol..

[15] Han R.L., Wang J., Zhang F.J., Zhao N., Gao B.L. (2019). Ultrasound risk assessment combined with molecular markers of galectin-3, c-met, hbme-1 and ck19 for diagnosis of malignant and benign thyroid nodules.. Pathol. Oncol. Res..

[16] Liu X., Liu Y. (2025). Expression patterns and diagnostic efficacy of cytokeratin 19 and galectin-3 in thyroid neoplasms.. Int. J. Clin. Exp. Pathol..

[17] El Demellawy D., Nasr A., Alowami S. (2008). Application of CD56, P63 and CK19 immunohistochemistry in the diagnosis of papillary carcinoma of the thyroid.. Diagn. Pathol..

[18] Na D.G., Baek J.H., Jung S.L., Kim J., Sung J.Y., Kim K.S., Lee J.H., Shin J.H., Choi Y.J., Ha E.J., Lim H.K., Kim S.J., Hahn S.Y., Lee K.H., Choi Y.J., Youn I., Kim Y.J., Ahn H.S., Ryu J.H., Baek S.M., Sim J.S., Jung C.K., Lee J.H. (2017). Core needle biopsy of the thyroid: 2016 consensus statement and recommendations from Korean society of thyroid radiology.. Korean J. Radiol..

[19] Su X., Yue C., Yang W., Ma B. (2024). A comparative analysis of core needle biopsy and repeat fine needle aspiration in patients with inconclusive initial cytology of thyroid nodules.. Front. Endocrinol..

[20] Vishwanath D., Shanmugam A., Sundaresh M., Hariharan A., Saraf S., Bahadur U., Veeramachaneni V., Chandrasekhar N., Pillai V V., Bushan V., Shetty V., Subramaniam N., Kuriakose M.A., Shivaprasad K.S., Khadilkar K., Suresh A., Sum S., Lakhsmikantha A., Rekha P.R., Zaidi S.N., Gupta V., Kannan S. (2022). Development of a low-cost NGS test for the evaluation of thyroid nodules.. Indian J. Surg. Oncol..

[21] Liu Q., Castelli M., Gattuso P., Prinz R.A. (1995). Simultaneous fine-needle aspiration and core-needle biopsy of thyroid nodules.. Am. Surg..

[22] Paja M., del Cura J.L., Zabala R., Korta I., Ugalde A., López J.I. (2019). Core-needle biopsy in thyroid nodules: Performance, accuracy, and complications.. Eur. Radiol..

[23] Jung C.K. (2023). Reevaluating diagnostic categories and associated malignancy risks in thyroid core needle biopsy.. J. Pathol. Transl. Med..

[24] Ha E.J., Baek J.H., Lee J.H., Kim J.K., Choi Y.J., Sung T.Y., Kim T.Y. (2017). Complications following US-guided core-needle biopsy for thyroid lesions: A retrospective study of 6,169 consecutive patients with 6,687 thyroid nodules.. Eur. Radiol..

[25] Nasrollah N., Trimboli P., Guidobaldi L., Cicciarella Modica D.D., Ventura C., Ramacciato G., Taccogna S., Romanelli F., Valabrega S., Crescenzi A. (2013). Thin core biopsy should help to discriminate thyroid nodules cytologically classified as indeterminate. A new sampling technique.. Endocrine.

[26] Jin Kim H., Koon Kim Y., Hoon Moon J., Young Choi J., Il Choi S. (2019). Thyroid core needle biopsy: Patients’ pain and satisfaction compared to fine needle aspiration.. Endocrine.

[27] Jung C.K., Baek J.H., Na D.G., Oh Y.L., Yi K.H., Kang H.C. (2020). 2019 Practice guidelines for thyroid core needle biopsy: A report of the Clinical Practice Guidelines Development Committee of the Korean Thyroid Association.. J. Pathol. Transl. Med..

[28] Joo L., Na D.G., Kim J., Seo H. (2022). Comparison of core needle biopsy and repeat fine-needle aspiration in avoiding diagnostic surgery for thyroid nodules initially diagnosed as atypia/follicular lesion of undetermined significance.. Korean J. Radiol..

[29] Hong M.J., Na D.G., Lee H. (2020). Diagnostic efficacy and safety of core needle biopsy as a first-line diagnostic method for thyroid nodules: A prospective cohort study.. Thyroid.

[30] Trimboli P., Giovanella L. (2018). Reliability of core needle biopsy as a second-line procedure in thyroid nodules with an indeterminate fine-needle aspiration report: A systematic review and meta-analysis.. Ultrasonography.

[31] Suh C.H., Baek J.H., Lee J.H., Choi Y.J., Kim K.W., Lee J., Chung K.W., Shong Y.K. (2016). The role of core-needle biopsy in the diagnosis of thyroid malignancy in 4580 patients with 4746 thyroid nodules: A systematic review and meta-analysis.. Endocrine.

[32] Hahn S.Y., Shin J.H., Lim H.K., Jung S.L. (2017). Follicular variant of papillary thyroid carcinoma: Comparison of ultrasound-guided core needle biopsy and ultrasound-guided fine needle aspiration in a multicentre study.. Clin. Endocrinol..

[33] Moon H.H., Chung S.R., Choi Y.J., Sung T.Y., Song D.E., Kim T.Y., Lee J.H., Baek J.H. (2024). The diagnostic role of repeated biopsy of thyroid nodules with atypia of undetermined significance with architectural atypia on core-needle biopsy.. Endocrinol. Metab..

[34] Cunha C., Mousinho F., Saraiva C., Sequeira Duarte J. (2023). Follicular lymphoma of the thyroid and the role of core needle biopsy.. Endocrinol. Diabetes Metab. Case Rep..

[35] Trimboli P., Nasrollah N., Amendola S., Crescenzi A., Guidobaldi L., Chiesa C., Maglio R., Nigri G., Pontecorvi A., Romanelli F., Giacomelli L., Valabrega S. (2015). A cost analysis of thyroid core needle biopsy *vs*. diagnostic surgery.. Gland Surg..

[36] Luo P., Ma W., Jiao D. (2024). Thyroid fine needle aspiration specimen adequacy: A noninferiority study and cost-effectiveness comparison of puncture needles.. Sci. Rep..

[37] Yoon J.H., Kim E.K., Kwak J.Y., Moon H.J. (2015). Effectiveness and limitations of core needle biopsy in the diagnosis of thyroid nodules: Review of current literature.. J. Pathol. Transl. Med..

[38] Mukherjee G., Biswas S., Haldar B., Dutta T. (2022). Correlation of fine needle aspiration cytology and cell block study of category III, IV, and V (TBSRTC-2017) thyroid lesions with special reference to cytokeratin-19 immunohistochemical staining.. Indian J. Pathol. Microbiol..

[39] Liberman E., Weidner N. (2000). Papillary and follicular neoplasms of the thyroid gland. Differential immunohistochemical staining with high-molecular-weight keratin and involucrin.. Appl. Immunohistochem. Mol. Morphol..

[40] Cheung C.C., Ezzat S., Freeman J.L., Rosen I.B., Asa S.L. (2001). Immunohistochemical diagnosis of papillary thyroid carcinoma.. Mod. Pathol..

[41] Zhu X., Sun T., Lu H., Zhou X., Lu Y., Cai X., Zhu X. (2010). Diagnostic significance of CK19, RET, galectin-3 and HBME-1 expression for papillary thyroid carcinoma.. J. Clin. Pathol..

[42] Elsers D., Hussein M., Osman M., Mohamed G., Hosny G. (2021). Challenge in the pathological diagnosis of the follicular- patterned thyroid lesions.. Asian Pac. J. Cancer Prev..

[43] Tastekin E., Keskin E., Can N., Canberk S., Mut A., Erdogan E., Asa N., Güldiken S., Sezer A., Azatcam M. (2019). CD56, CD57, HBME1, CK19, Galectin-3 and p63 immunohistochemical stains in differentiating diagnosis of thyroid benign/malign lesions and NIFTP.. Pol. J. Pathol..

[44] Arcolia V., Journe F., Renaud F., Leteurtre E., Gabius H.J., Remmelink M., Saussez S. (2017). Combination of galectin-3, CK19 and HBME-1 immunostaining improves the diagnosis of thyroid cancer.. Oncol. Lett..

[45] Huang L., Wang X., Huang X., Gui H., Li Y., Chen Q., Liu D., Liu L. (2018). Diagnostic significance of CK19, galectin-3, CD56, TPO and Ki67 expression and BRAF mutation in papillary thyroid carcinoma.. Oncol. Lett..

[46] Barroeta J.E., Baloch Z.W., Lal P., Pasha T.L., Zhang P.J., LiVolsi V.A. (2006). Diagnostic value of differential expression of CK19, Galectin-3, HBME-1, ERK, RET, and p16 in benign and malignant follicular-derived lesions of the thyroid: An immunohistochemical tissue microarray analysis.. Endocr. Pathol..

[47] Onieva Carbajo R., Ferreres Piñas J.C., Alcalà Lorente M., Capel Flores I., Cano Palomares A., Bella Burgos B., Guirao Garriga F.J., Pérez-Riverola V., Escoda Giralt M.R., Barcons Vilaplana S., Monmany Badia R., El Hamshari Rebollo K., Padilla Navas C., Bella Cueto M.R. (2025). Usefulness of CD56, BRAF and CD15 in the differential diagnosis of thyroid lesions with nuclear features of papillary carcinoma.. Pathol. Res. Pract..

[48] Erdogan-Durmus S., Ozcan D., Yarikkaya E., Kurt A., Arslan A. (2016). CD56, HBME-1 and cytokeratin 19 expressions in papillary thyroid carcinoma and nodular thyroid lesions.. J. Res. Med. Sci..

[49] Pyo J.S., Kim D.H., Yang J. (2018). Diagnostic value of CD56 immunohistochemistry in thyroid lesions.. Int. J. Biol. Markers.

[50] Wang X., Zhou H., Iqbal M.A., Pan D., Zuo J., Moazzam N.F., Zhang J., Sun H. (2025). A case series of ultrasound and pathological assessment of follicular thyroid tumors: Addressing indeterminate malignancy.. Medicine.

[51] Chiu C.G., Strugnell S.S., Griffith O.L., Jones S.J.M., Gown A.M., Walker B., Nabi I.R., Wiseman S.M. (2010). Diagnostic utility of galectin-3 in thyroid cancer.. Am. J. Pathol..

[52] Collet J.F., Hurbain I., Prengel C., Utzmann O., Scetbon F., Bernaudin J.F., Fajac A. (2005). Galectin-3 immunodetection in follicular thyroid neoplasms: A prospective study on fine-needle aspiration samples.. Br. J. Cancer.

[53] Trimboli P., Guidobaldi L., Amendola S., Nasrollah N., Romanelli F., Attanasio D., Ramacciato G., Saggiorato E., Valabrega S., Crescenzi A. (2016). Galectin-3 and HBME-1 improve the accuracy of core biopsy in indeterminate thyroid nodules.. Endocrine.

[54] Trimboli P., Virili C., Romanelli F., Crescenzi A., Giovanella L. (2017). Galectin-3 performance in histologic and cytologic assessment of thyroid nodules: A systematic review and meta-analysis.. Int. J. Mol. Sci..

[55] Bartolazzi A., Orlandi F., Saggiorato E., Volante M., Arecco F., Rossetto R., Palestini N., Ghigo E., Papotti M., Bussolati G., Martegani M.P., Pantellini F., Carpi A., Giovagnoli M.R., Monti S., Toscano V., Sciacchitano S., Pennelli G.M., Mian C., Pelizzo M.R., Rugge M., Troncone G., Palombini L., Chiappetta G., Botti G., Vecchione A., Bellocco R. (2008). Galectin-3-expression analysis in the surgical selection of follicular thyroid nodules with indeterminate fine-needle aspiration cytology: A prospective multicentre study.. Lancet Oncol..

[56] Zargari N., Mokhtari M. (2019). Evaluation of diagnostic utility of immunohistochemistry markers of trop-2 and hbme-1 in the diagnosis of thyroid carcinoma.. Eur. Thyroid J..

[57] Abu-Seadah S., Attiah S., Ali M., Shams El-Din M., El-Kholy M. (2023). Immunohistochemical expression of HBME-1 and TROP-2 in some follicular-derived thyroid lesions.. Asian Pac. J. Cancer Prev..

[58] de Matos L.L., Del Giglio A.B., Matsubayashi C.O., de Lima Farah M., Del Giglio A., da Silva Pinhal M.A. (2012). Expression of CK-19, galectin-3 and hbme-1 in the differentiation of thyroid lesions: Systematic review and diagnostic meta-analysis.. Diagn. Pathol..

[59] Cazzaniga G., Seminati D., Smith A., Piga I., Capitoli G., Garancini M., L’Imperio V., Fusco N., Pagni F. (2022). Lights on HBME-1: The elusive biomarker in thyroid cancer pathology.. J. Clin. Pathol..

[60] Kong J.S., Kim H.J., Kim M.J., Kim A., Lee D., Han K., Park S., Koh J.S., Myung J.K. (2018). The significance of TROP2 expression in predicting BRAF mutations in papillary thyroid carcinoma.. J. Pathol. Transl. Med..

[61] Wen Y., Ouyang D., Zou Q., Chen Q., Luo N., He H., Anwar M., Yi W. (2022). A literature review of the promising future of *TROP2*: A potential drug therapy target.. Ann. Transl. Med..

[62] Nechifor-Boila A., Borda A., Sassolas G., Hafdi-Nejjari Z., Borson-Chazot F., Lifante J.C., Sturm N., Lavérriere M.H., Berger N., Decaussin-Petrucci M. (2013). Immunohistochemical markers in the diagnosis of papillary thyroid carcinomas: The promising role of combined immunostaining using HBME-1 and CD56.. Pathol. Res. Pract..

[63] Unger P., Ewart M., Wang B.Y., Gan L., Kohtz D.S., Burstein D.E. (2003). Expression of p63 in papillary thyroid carcinoma and in Hashimoto’s thyroiditis: A pathobiologic link?. Hum. Pathol..

[64] Jeong J.Y., Jung J.H., Park J.Y. (2016). Expression and diagnostic availability of p63 and CD56 in papillary thyroid carcinoma.. Int J Clin Exp Pathol.

[65] Trimboli P., Nasrollah N., Guidobaldi L., Taccogna S., Cicciarella Modica D.D., Amendola S., Romanelli F., Lenzi A., Nigri G., Centanni M., Giovanella L., Valabrega S., Crescenzi A. (2014). The use of core needle biopsy as first-line in diagnosis of thyroid nodules reduces false negative and inconclusive data reported by fine-needle aspiration.. World J. Surg. Oncol..

[66] Song S., Kim H., Ahn S.H. (2019). Role of immunohistochemistry in fine needle aspiration and core needle biopsy of thyroid nodules.. Clin. Exp. Otorhinolaryngol..

[67] Xiong Y., Li X., Liang L., Li D., Yan L., Xueying L., Di J., Li T. (2021). Application of biomarkers in the diagnosis of uncertain samples of core needle biopsy of thyroid nodules.. Virchows Arch..

[68] Xin Y., Guan D., Meng K., Lv Z., Chen B. (2017). Diagnostic accuracy of CK-19, Galectin-3 and HBME-1 on papillary thyroid carcinoma: A meta-analysis.. Int. J. Clin. Exp. Pathol..

[69] Mai K.T., Yazdi H.M., Commons A.S., Perkins D.G., MacDonald L. (1999). Neoplastic non-papillary thyroid carcinoma lesions with a fine chromatin pattern.. Pathol. Int..

[70] Baloch Z.W., Gupta P.K., Yu G.H., Sack M.J., LiVolsi V.A. (1999). Follicular variant of papillary carcinoma. Cytologic and histologic correlation.. Am. J. Clin. Pathol..

[71] Park Y.J., Kwak S.H., Kim D.C., Kim H., Choe G., Park D.J., Jang H.C., Park S.H., Cho B.Y., Park S.Y. (2007). Diagnostic value of galectin-3, HBME-1, cytokeratin 19, high molecular weight cytokeratin, cyclin D1 and p27(kip1) in the differential diagnosis of thyroid nodules.. J. Korean Med. Sci..

[72] Madani S.H., Shahebrahimi K., Fazaeli A., Khazaei S., Kanani M., Keshavarz A. (2013). Diagnostic value of CD56 and nm23 markers in papillary thyroid carcinoma.. Indian J. Pathol. Microbiol..

[73] Shin M.K., Kim J.W., Ju Y.S. (2011). CD56 and high molecular weight cytokeratin as diagnostic markers of papillary thyroid carcinoma.. Korean J. Pathol..

[74] Massa F., Caraci P., Sapino A., De Rosa G., Volante M., Papotti M. (2021). Outcome and diagnostic reproducibility of the thyroid cytology “indeterminate categories” SIAPEC/SIE 2014 in a consecutive series of 302 cases.. J. Endocrinol. Invest..

[75] Cozzani F., Bettini D., Rossini M., Bonati E., Nuzzo S., Loderer T., Pedrazzi G., Zaccaroni A., Del Rio P. (2021). Thyroid nodules with indeterminate cytology: Association between nodule size, histopathological characteristics and clinical outcome in differentiated thyroid carcinomas — a multicenter retrospective cohort study on 761 patients.. Updates Surg..

[76] Torregrossa L., Poma A.M., Macerola E., Rago T., Vignali P., Romani R., Proietti A., Di Stefano I., Scuotri G., Ugolini C., Basolo A., Antonelli A., Materazzi G., Santini F., Basolo F. (2022). The Italian C onsensus for the Classification and Reporting of Thyroid Cytology: Cytohistologic and molecular correlations on 37,371 nodules from a single institution.. Cancer Cytopathol..

[77] Trimboli P., Ferrarazzo G., Cappelli C., Piccardo A., Castellana M., Barizzi J. (2022). Thyroid nodules with indeterminate fnac according to the italian classification system: Prevalence, rate of operation, and impact on risk of malignancy. an updated systematic review and meta-analysis.. Endocr. Pathol..

[78] Guo M., Sun Y., Wei Y., Xu J., Zhang C. (2024). Advances in targeted therapy and biomarker research in thyroid cancer.. Front. Endocrinol..

